# Fish Gill-Inspired Bidirectional Porous Polysaccharide Aerogels for Micro/Nanoplastics Removal

**DOI:** 10.1021/acsami.5c18203

**Published:** 2025-11-11

**Authors:** Yunchong Yang, Weijia Yan, Jun Ma, David Carmona, Chunka Zhou, Elise Nguyen, Jingjing Qiu

**Affiliations:** † Department of Materials Science and Engineering, 14736 Texas A&M University, 3003 TAMU, College Station, Texas 77843, United States; ‡ Department of Mechanical Engineering, 14736 Texas A&M University, 3123 TAMU, College Station, Texas 77843, United States; § Department of Biomedical Engineering, 14736 Texas A&M University, 3131 TAMU, College Station, Texas 77843, United States; ∥ Department of Mechanical Engineering, Texas A&M University at Qatar, PO Box 23874, Education City, Doha, Qatar

**Keywords:** micro/nanoplastics removal, polysaccharide, bidirectional aerogel, fish gill inspiration, sustainability, continuous-flow adsorption, antibacterial

## Abstract

Micro/nanoplastics (MNPs) pose a threat to freshwater ecosystems and human health due to their intrinsic toxicity, leaching of harmful degradation byproducts, and transport of pollutants through food chains. However, the efficient removal of MNPs using conventional methods such as filtration, flocculation, and biodegradation remains a significant challenge. Inspired by the structure of fish gills, we developed a sustainable bidirectionally porous polysaccharide-based aerogel composed of chitosan (CS), cellulose nanofibers (CNFs), and polydopamine (PDA) for the effective removal of MNPs. The aerogel demonstrated outstanding adsorption capacities exceeding 300 mg/g for various MNP types, including carboxylated polystyrene (PS-COOH), PS, poly­(methyl methacrylate) (PMMA), polyethylene (PE), and polypropylene (PP). In particular, the bidirectionally oriented porous structure achieved an adsorption capacity of more than 9 times greater than that of randomly oriented aerogels. Molecular dynamics (MD) simulations revealed that this high adsorption performance is attributed to strong multimodal interactions between the aerogel and MNPs, including electrostatic interactions, van der Waals (vdW) forces, hydrogen bonding, and π–π interactions. A benchtop continuous-flow adsorption column system was constructed, enabling an MNP removal efficiency of over 96% for 1 L of MNP-contaminated water within four purification cycles and a total of 20 min. Additionally, the aerogels exhibited excellent antibacterial activity against *E. coli* in acidic environments. Overall, with its large adsorption capacity, high water flux, and remarkable antibacterial properties, these bioinspired aerogels offer a scalable and sustainable solution for efficient MNP remediation from wastewater streams. This work uncovers how directional pore alignment, tailored surface chemistry, and multimodal interactions synergistically enhance the adsorption of diverse MNP species.

## Graphical abstract

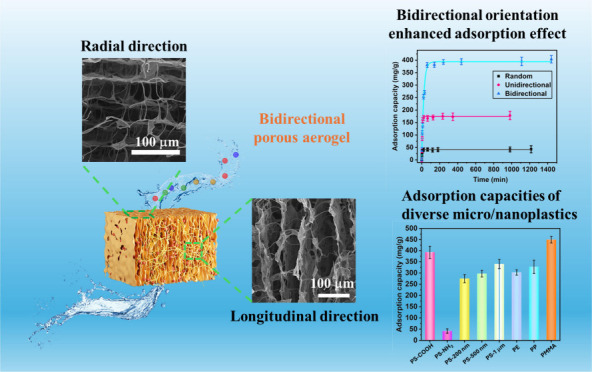

## 1 Introduction

Micro/nanoplastics (MNPs) contain nanoplastics (NPs, <1 μm) and microplastics (MPs, <5 mm) that originate from various sources, including degraded plastic bottles, synthetic textiles, and personal care products. In 2019, global plastic waste generation was estimated at 353 million tons, with 79 million tons improperly managed and subsequently entering aquatic environments due to inadequate disposal practices,^1^ such as polypropylene (PP), polystyrene (PS), polyethylene (PE), polyethylene terephthalate (PET), and poly­(methyl methacrylate) (PMMA), etc. These MNPs are resistant to complete degradation in natural aqueous conditions, allowing them to carry toxic chemicals and readily transfer the toxins through the food chain, resulting in contaminated freshwater and potential health risks to humans.^2^ Therefore, addressing MNPs pollution in natural aquatic environments has become an increasingly urgent task.

Conventional water remediation techniques such as membrane filtration,^3^ flocculation,^4^ coagulation,^5^ biodegradation,^6^ and electrophoresis^7^ have inherent limitations in removing MNP pollutants, including high cost, energy consumption, clogging, and secondary pollution. In contrast, adsorption has emerged as a promising alternative due to its simplicity, safety, low energy consumption, and cost-effectiveness.^8^ Despite intensive studies on biochar,^9^ activated carbon materials,^10^ graphene oxide,^11^ and metal–organic framework^12^ for solid-phase wastewater treatment, these materials generally suffer from complex synthesis, intensive chemical usage, prolonged settling duration, and secondary waste generation.

In contrast, polysaccharide-based adsorbents represent a sustainable class of adsorbents that combine strong pollutant affinity, environmental safety, biodegradability, and affordability with minimized risk of secondary pollution.^13^ The abundant surface groups (e.g., −OH, −NH_2_, −COOH) on polysaccharides (e.g., chitosan, cellulose, chitin^14^) and polysaccharide-rich natural biomaterials (e.g., fungal mycelium^15^ and lily bulbs^16^) enable facile chemical modification for enhanced MNP affinity and loading capacity. Chitosan (CS), derived from crustacean shells (e.g., shrimp, crab) or fungal cell walls, contains abundant amino and hydroxyl groups that facilitate electrostatic interactions with negatively charged particles in aqueous environments. Similarly, cellulose nanofibrils (CNF), extracted from plant biomass (wood, agricultural residues), possess high aspect ratios and hydroxyl-rich surfaces, supporting the assembly of porous aerogels with high strength and controllable surface chemistry. Previous studies demonstrated that CS- and CNF-based aerogels achieve high removal efficiencies for MNPs in aquatic environments.^17–20^ However, their synthesis often requires elaborate procedures and toxic modifiers (e.g., glutaraldehyde, urea), leading to increased cost, reduced efficiency, and potential secondary contamination.^21–23^ Furthermore, conventional foams/aerogels have randomly oriented (isotropic) microporous structures,^24,25^ which limit their mechanical properties, mass transport, and adsorption efficiency for MNP extraction, especially for the nanosized plastics <1 μm. As a result, they exhibit insufficient binding affinity toward hydrophobic MNPs, such as PS, PE, PP, and PMMA. Therefore, the growing efforts focus on sustainable polysaccharide-based aerogels that combine multiple binding mechanisms, circumvent the use of toxic or persistent modifiers, and employ facile fabrication approaches to create anisotropic porous architectures for broad-spectrum MNP capture.

Fish gill rakers can capture particles that are much smaller than their inter-raker spacing through coupled hydrodynamic and physicochemical mechanisms. In addition to size-exclusion and flow-induced filtration, a mucopolysaccharide gel coating on the rakers’ surface enables adhesive/adsorptive capture via synergistic electrostatic, hydrogen-bonding, and hydrophobic interactions. Furthermore, the bidirectionally oriented lamella on gill filaments allows a rapid water flow through fish gills. Inspired by the fish gill structures, as illustrated in Scheme 1a, a polysaccharide-based aerogel was fabricated in this study via bidirectional freeze-drying. Briefly, the bidirectional porous aerogel Bi-CS-CNF-PDA was assembled from CS, CNF, and polydopamine (PDA) for efficient MNPs removal in aquatic environments. Bidirectional freeze-drying method was used to form 3D interpenetrating microporous structures with enhanced mechanical strength, resilience, and adsorption capacity,^26–28^ as shown in Scheme 1b. As-fabricated aerogels indicated highly porous, lightweight, and mechanically stable microstructures that leverage multimodal interactions for enhanced binding affinity toward MNPs. As illustrated in Scheme 1c, with the introduction of abundant catechol and amine functional groups by PDA immobilization, bidirectional aerogels exhibited strong adhesive properties and broad-spectrum pollutants capture through electrostatic attraction, π–π stacking, hydrogen bonding, and hydrophobic interactions.^22,29–34^ As-synthesized Bi-CS-CNF-PDA aerogels demonstrated excellent adsorption capacity of over 300 mg/g toward PS-COOH, PS, PE, PP, and PMMA with different particle sizes. Moreover, a benchtop continuous-flow adsorption system was developed to validate the efficient adsorptive filtration of MNPs and enhanced mass transport for the MNP-contaminated wastewater streams, highlighting its feasibility for large-scale and continuous wastewater treatment processes. Finally, the sustainable CS-CNF-PDA aerogels indicated excellent antibacterial performance against *E. coli*, attributed to the synergistic effects of CS’s cationic nature and the reactive functional groups of PDA. Such antibacterial performance suppresses biofouling and microbial degradation, thereby enhancing the aerogel’s operational stability and enabling prolonged reuse during wastewater treatment.

**Scheme 1:**
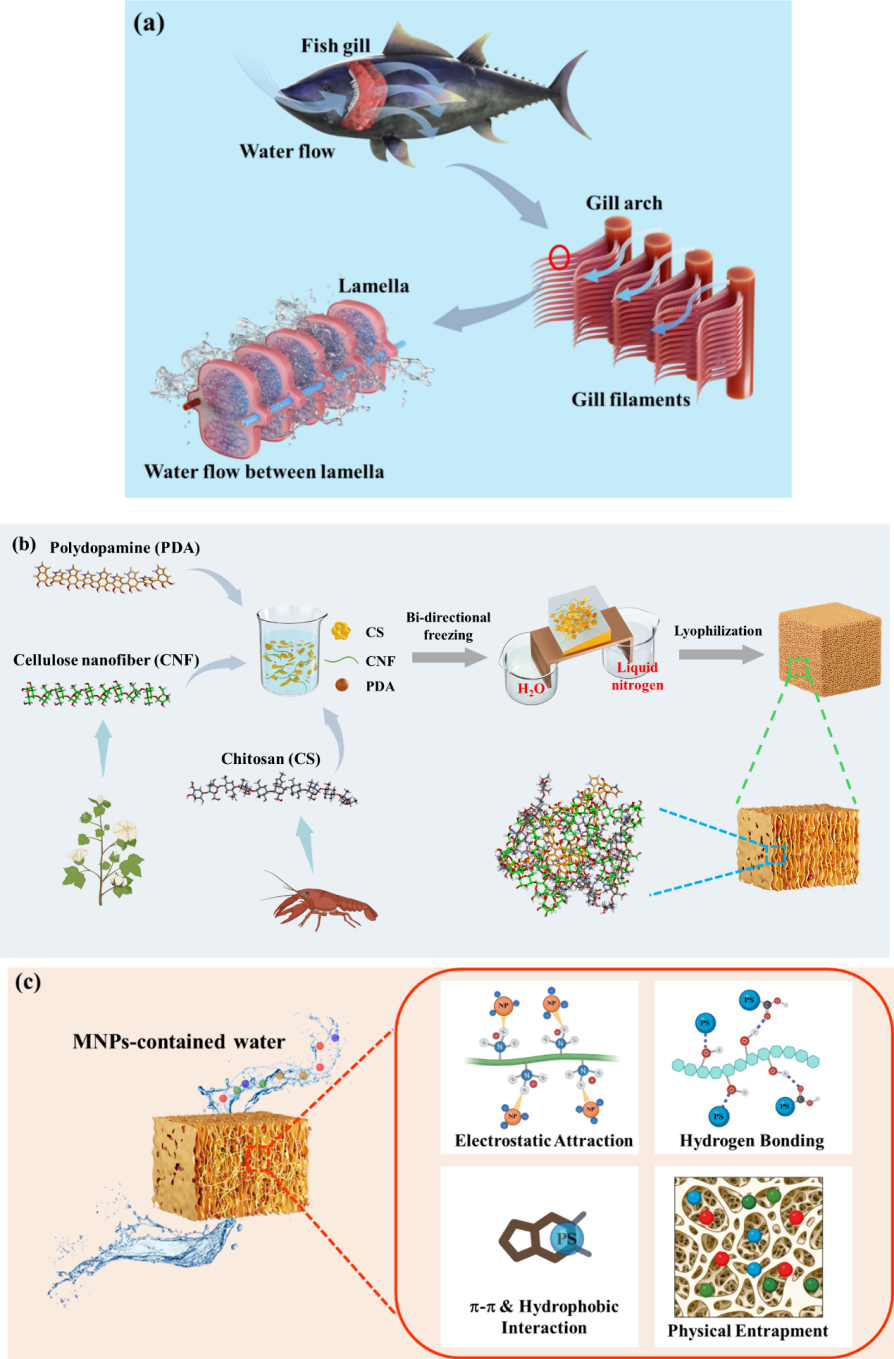
Schematic Illustrations of (a) Multiscale Architecture of Fish Gills, (b) Fabrication Procedures for Bi-CS-CNF-PDA Aerogel, and (c) Micro/Nanoplastic (MNP) Removal by an Adsorptive Filtration System through Multiple Intermolecular Interactions

## 2 Experimental Section

### 2.1 Materials and Chemicals

Cellulose nanofibers (CNF) were purchased from CELLULOSE LAB (Canada). Chitosan (deacetylation ≥ 75%) and Dopamine hydrochloride (>98%, TLC) were purchased from Sigma-Aldrich. Red fluorescent PS-COOH (500 nm), PS-NH_2_ (530 nm) (505–545 nm excitation, 560–630 nm emission), and green-fluorescent PS MNPs (200, 500, 1000 nm) (488 nm excitation, 509 nm emission) were obtained from Magsphere Inc. (CA, USA). Red fluorescent PMMA and PE MNPs (500 nm) (excitation at 620 nm, emission at 680 nm) were purchased from Abvigen Inc. (NJ, USA). These MNPs were used to prepare wastewater contaminated with MNPs.

### 2.2 Synthesis of Polydopamine

0.1 g of dopamine hydrochloride and 0.2 mL ammonia were dissolved in 15 mL of ethanol solution with magnetic stirring for 1 h at room temperature. Then, acetic acid was added to the solution until the pH was 7.0. In this process, polydopamine particles were synthesized by the self-polymerization of dopamine hydrochloride under alkaline aerobic conditions.

### 2.3 Preparation of Bidirectional CS-CNF-PDA Aerogels

1.5 mL of CS solution (1 wt %) and 1.5 mL of CNF suspension (1 wt %) were mixed in a glass vial with a volume ratio of 1:1. Then, 90 μL of PDA solution was mixed with the CS and CNF solution at 1000 rpm for 60 min at room temperature. The materials were further dispersed by bath sonication for 30 min to obtain a well-mixed viscous hydrogel solution. The mixture solution was then placed on the top of a bidirectional freezing setup, as shown in Figure 1a. The setup consisted of a U-shaped copper bracket with one end immersed in liquid nitrogen and the other end immersed in icy water, creating a temperature gradient across the middle web section of the copper bracket, where a vial containing the hydrogel solution was placed. The second temperature gradient was imposed along the longitudinal direction of the vial, extending from the freezing zone at the bottom to ambient temperature on the top. The hydrogel solution was placed on the copper plate for 10 min until it was completely frozen. The frozen sample was then retained in a freezer at −20 °C overnight and subsequently freeze-dried for 24 h under −54 °C to obtain the Bi-CS-CNF-PDA aerogel.

**Figure 1:**
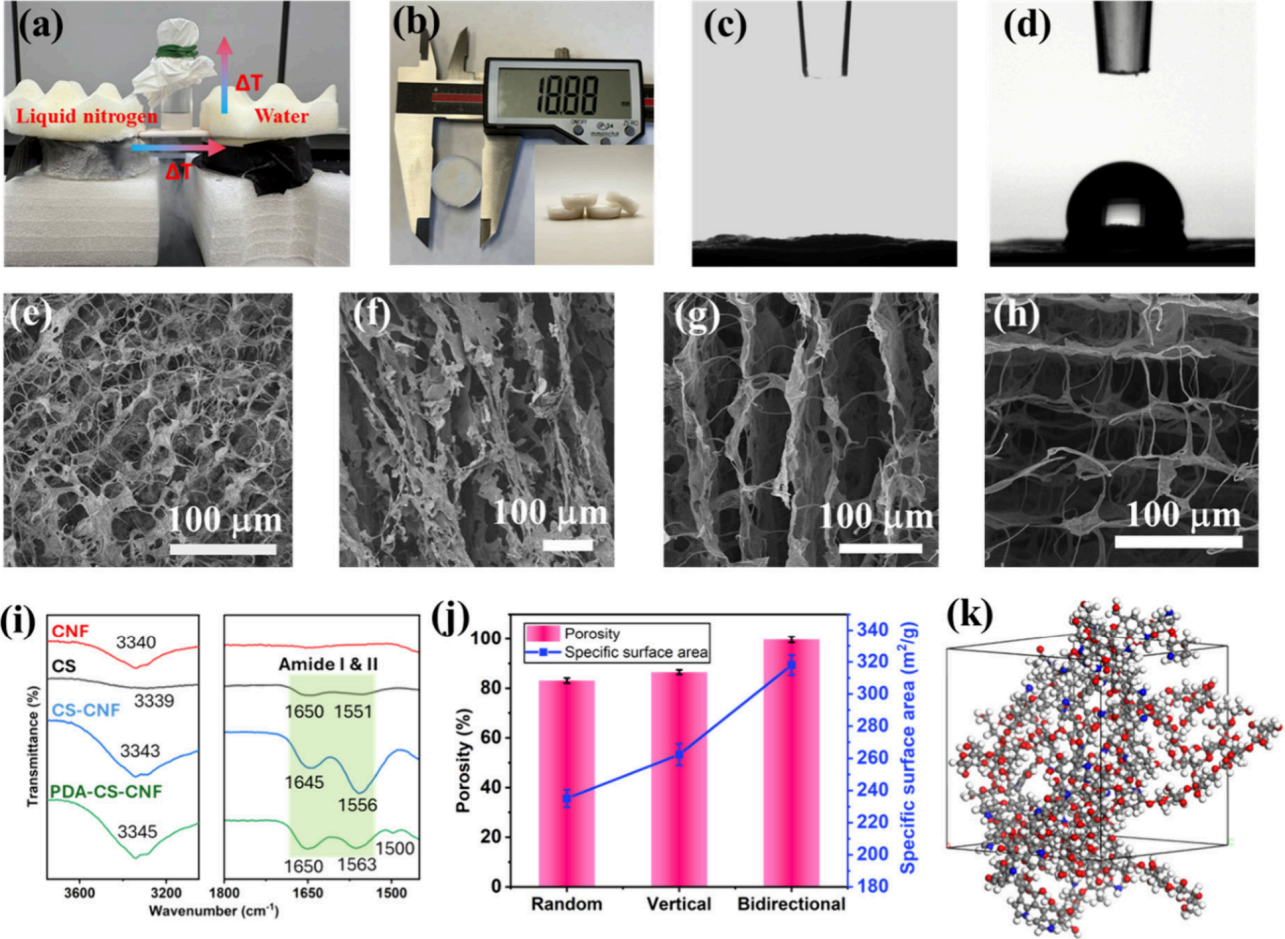
Fabrication and characterization of bidirectional CS-CNF-PDA aerogels. (a) The bidirectional freeze casting setup for the preparation of Bi-CS-CNF-PDA aerogels. (b) Photograph of the synthesized free-standing Bi-CS-CNF-PDA aerogels. (c, d) Water contact angles of the synthesized CS-CNF and CS-CNF-PDA aerogels. SEM images of (e) random CS-CNF-PDA, (f) unidirectionally oriented CS-CNF-PDA, and (g and h) longitudinal and radial directions of Bi-CS-CNF-PDA. (i) FT-IR spectra of CNF, CS, CS-CNF, and PDA-CS-CNF aerogels. (j) Porosities and specific surface areas of CS-CNF-PDA aerogels with different orientations (random, unidirectional, and bidirectional). (k) Molecular model of the CS-CNF-PDA aerogel. Error bars represent the standard deviation of three independent measurements.

### 2.4 Characterization

The morphology of the aerogels was characterized using a JEOL JSM-7500F field-emission scanning electron microscope (FE-SEM) instrument. Attenuated total reflection Fourier transform-infrared (ATR-FTIR) spectra were recorded on an ALFHA-Platinum (Bruker). Water contact angle analysis was performed using an Optical Tensiometer (Theta Flex, Biolin Scientific, Sweden). Size distributions and surface zeta potential of nanoparticles in solutions were evaluated by a Zetasizer Nano ZS dynamic light scattering (DLS) (Malvern Panalytical, UK). Nanoparticle tracking analysis (NTA) measurements were conducted using a Nanosight LM10 Instrument (Malvern Panalytical, UK). Uniaxial compression tests of the aerogels were performed using a Dynamic Mechanical Analysis (DMA) instrument (DMA 850, TA Instruments) at a strain rate of 10% per minute at room temperature.

### 2.5 Porosity Measurement

The diameter and height of the aerogel were measured by a vernier caliper, and the volume of the aerogel was calculated. Then, the aerogel was weighed as m_1_ and soaked in isopropanol for 30 min until it was saturated, after which it was weighed again as m_2_. The equation for porosity calculation was listed in equation 1:^35^

1 $$Porosity\;\left(\%\right)=\frac{m_{2}-m_{1}}{\rho \times V}\times 100\%$$

Where m_2_ (g) and m_1_ (g) are the saturated aerogel mass and dry aerogel mass, respectively; ρ (g/cm^3^) is the density of isopropanol; and V (cm^3^) is the original bulk volume of the samples.

### 2.6 MNP Adsorption Performance Evaluation

The adsorption properties of as-fabricated CS-CNF-PDA aerogels toward fluorescence-labeled PS-COOH microsphere with a diameter of 500 nm were studied. To explore the kinetics of PS-COOH adsorption on CS-CNF-PDA, 3 mg of aerogel was immersed in the PS-COOH suspension (0.5 mg/mL, 3 mL), which was then shaken in an incubating orbital shaker (VWR, USA) with 120 rpm at 25 °C and pH 7 for different durations (0, 2, 5, 10, 20, 30, 40, 60, 120, 240, 360, 480, and 1440 min). The suspension concentrations were calculated based on the fluorescence intensity, as detected by an Infinite 200 Pro microplate reader (Tecan Ltd., Switzerland) before and after adsorption. By preparing a series of standard solutions containing PS-COOH, the standard curve function shown in Figure S7 was determined by measuring their fluorescence intensity under an excitation and emission wavelength of 508 and 595 nm, respectively. The standard curve function was then used to relate the fluorescence intensity of the suspension to its MNP concentrations at different adsorption times. After that, the effect of pH value on PS-COOH adsorption by the bidirectional aerogel was studied over a range of pH values across 3 to 11 using 0.1 M HCl and NaOH solutions. Furthermore, the effect of initial concentration on adsorption was studied by preparing PS-COOH suspensions with initial concentrations of 0.05, 0.1, 0.2, 0.5, 1, 1.5, and 2 mg/mL under neutral pH conditions for 240 min at 25 °C. Under optimal pH value and initial concentration, the adsorption effects of the bidirectional CS-CNF-PDA aerogels toward fluorescent PS MNPs with different particle sizes (200, 500, and 1000 nm) and different charges (PS-NH_2_) were studied with an initial concentration of 0.5 mg/mL for 240 min at 25 °C. Subsequently, the effects of different MNP species, including 500 nm fluorescent PE, PP, and PMMA particles, were also evaluated and compared. The adsorption capacity of the CS-CNF-PDA aerogels was calculated through equation 2:^36^

2 $$Adsorption\;capacity\;\left(q\right)=\frac{\left(C_{0}-C\right)\times V}{m}$$

where q (mg/g) represents the mass of MNPs adsorbed per unit mass of aerogels; C_0_ and C (mg/mL) represent the initial concentration and the concentration after adsorption; V (mL) is the volume of the MNP suspension; and m (mg) indicates the weight of the dry aerogel.

### 2.7 MD Simulation

MD simulation was performed using BIOVIA Materials Studio 2023’s Amorphous Cell Module. THE COMPASS III force field was used to predict the molecular interactions between aerogels and diverse MNPs in solvent. The solution environment was set to be acidic by adding 1000 water molecules along with hydronium ions in the amorphous cell. MD simulation was carried out in the canonical (NVT) ensemble using V-rescale thermostats with a time step of 1 ps at 298 K. The total dynamic simulation time was set as 100 ps. Long-range electrostatic interactions were calculated via Ewald summation with a real-space cutoff distance of 1.2 nm. The systems were then subjected to 100 ps of MD simulation. The final structures obtained from the simulations were used for subsequent energy calculation and analysis. The binding energy (ΔE) between the CS-CNF-PDA and MNPs was calculated. ΔE is defined as the energy variation before and after aerogel–MNPs binding as shown in equation 3:^15^

3 $$\Delta E=E_{\mathrm{aerogel}+\mathrm{MNPs}}-\left(E_{\mathrm{aerogel}}+E_{\mathrm{MNPs}}\right)$$

where *E*_aerogel+MNPs_, *E*_aerogel_, and *E*_MNPs_ represent the total electronic energies of the aerogel–MNPs complex, the isolated aerogel, and the isolated MNPs, respectively.

### 2.8 Bench-Top Continuous-Flow Adsorptive Filtration System for MNP-Contaminated Water Treatment

A benchtop recirculating adsorptive filtration column for MNP-contaminated water treatment was assembled using a vertically mounted glass column (30 mm inner diameter, 10 in. in length) packed with aerogel adsorbents. Raw wastewater was provided by the Gulf Coast Water Authority as the matrix, into which additional fluorescent MNPs were introduced to simulate MNP-contaminated water. A mini submersible water pump delivered influent (MNP-contaminated water) from a reservoir at a controlled flow rate through the column from top to bottom. Effluent exiting the column was collected and recirculated back to the reservoir for subsequent treatment cycles, simulating multiple-cycle treatment. The system allows for continuous monitoring of adsorption efficiency over time. Fluorescence intensity measurements were used to quantify the removal of MNPs after each cycle.

### 2.9 Antibacterial Property Characterization

*Escherichia coli* (Sigma-Aldrich WDCM00) was selected to determine the antibacterial activity of CS-CNF-PDA aerogel. *E. coli.* was precultured in tryptic soy broth (TSB) at 37 °C with shaking at 55 rpm for 24 h from a single colony grown on a Petri dish. After incubation, 200 μL of the bacterial culture was transferred into a 24-well transparent plate and then diluted to a final volume of 2 mL with TSB adjusted to pH 6. The initial bacteria concentration was standardized to 5 × 10^4^ CFU/mL. Bacterial growth was monitored at 0, 1, 2, 4, 6, and 12 h by measuring the optical density at 600 nm (OD_600_). The medium with only TSB was used as controls (without aerogel) at pH 6.0 and pH 7.3 in the other two wells. Bacterial viability was further evaluated using a Live/Dead cell imaging kit (Thermo Fisher Scientific). Stained suspensions were observed under a fluorescence microscope, where live and dead cells were visualized as green and red, respectively.

## 3 Results and Discussion

### 3.1 Fabrication and Characterization of Fish Gill-Inspired Bidirectional CS-CNF-PDA Aerogels

The setup of bidirectional freezing is shown in Figure 1a. In this process, horizontal and vertical temperature gradients are simultaneously maintained for ice template growth. The polymer chains in the hydrogel solution are expelled into the space between adjacent ice crystals and assembled into parallel lamella.^37^ Subsequently, ice is removed via sublimation under vacuum, leaving highly interconnected porous microstructures that closely mimic the anatomy of lamella on gill filaments. The porous Bi-CS-CNF-PDA foam was obtained with a density of 0.012 g/cm^3^ (Figure 1b), which is a bit higher than that of the Bi-CS-CNF foam (0.009 g/cm^3^) due to the introduction of PDA. According to the water contact angle analysis, the CS-CNF aerogel was superhydrophilic with a contact angle approaching 0° (Figure 1c). However, it turned to be hydrophobic after PDA was introduced, with a water contact angle of 96.83° (Figure 1d), furthermore demonstrating the successful modification in surface chemistry of the CS-CNF aerogel. SEM images of the Bi-CS-CNF-PDA aerogel are shown in Figures 1g and 1h. Compared to randomly and unidirectionally oriented aerogels (Figure 1e and 1f), bidirectional aerogels exhibit two orthogonally oriented long-range lamellar channels with CS nanosheets along both longitudinal and radial directions, and an interlayer spacing of ∼ 65 μm. This structural characteristic endows the bidirectional aerogel with higher compressive strength, as shown in Figure S1. Lamellar CS serves as the primary framework in the porous architectures, providing the desired bulk integrity and shape stability. Meanwhile, CNF, with an ultrahigh aspect ratio, is interspersed on and between pore walls, forming struts that enhance the aerogel’s strength and toughness. Such bidirectional porous networks provide rapid fluid transport channels while the interconnected junctions offer mechanical robustness and reduced clogging. The observed results validated the successful fabrication of bidirectional channels and established a structural foundation for the adsorption of MNPs.

In addition to the mechanical reinforcement from CNF, the interactions between CS and CNF are also essential for the stabilization of the porous structure of this bidirectional biomass foam. The FT-IR spectra (Figure 1i) in as-synthesized CS-CNF foam reveal that the peaks of −OH stretching vibration (3339 cm^–1^), C=O stretching vibration in the amide I region (1650 cm^–1^), and N–H at the amide II region (1551 cm^–1^) shifted to 3343, 1645, and 1556 cm^–1^, respectively. These changes are attributed to the formation of intramolecular and intermolecular hydrogen bonds among −OH and -NH_2_ and -NHCO- functional groups. The emergence of a new peak at 1500 cm^–1^ in CS-CNF-PDA, attributed to the C=C/C=N vibration, verifies the successful synthesis of CS-CNF-PDA. As shown in Figure 1j, the porosity of Bi-CS-CNF-PDA was enhanced to 99.7%, in contrast to 79.7% for the random CS-CNF-PDA. The incorporation of directional structures increased the specific surface area from 235.05 m^2^/g in the random aerogel to 262.34 and 318.09 m^2^/g in the unidirectional and the bidirectional aerogels, respectively.

MD simulations were conducted for the CS-CNF-PDA composite under hydronium ions/H_2_O conditions to evaluate the structural stability. As shown in the energy profile (Figure S2), after ∼ 10 ps, the potential, kinetic, and nonbonded energies converged and stabilized with slight fluctuations, while the total energy remained constant for the rest of the simulation. This indicates that the CS-CNF-PDA composite has reached a thermodynamic equilibrium state, demonstrating good structural stability with no evidence of phase separation or molecular collapse during the MD simulation. Figure 1k shows the equilibrated configuration of the composite, illustrating a densely cross-linked and compactly packed network resulting from extensive interactions among the CS, CNF, and PDA chains.^38^ The abundant amine functional groups on CS and hydroxyl groups on CNF establish strong hydrogen bonds with nearby functional groups and active atoms. At the same time, aromatic rings in PDA enable π–π stacking interactions. These multimodal interactions endow the aerogel with long-term integrity in aqueous environments, making it a robust adsorbent for MNP removal.

### 3.2 Adsorption Performance Analysis

The adsorption performance of the synthesized Bi-CS-CNF-PDA foam toward the PS-COOH MNPs (500 nm) was studied. The size distribution of PS-COOH particles was characterized by the DLS technique, as shown in Figure S3. Figure 2a exhibits the morphology of the aerogel after adsorption of PS-COOH, where the bidirectional microstructure can be clearly observed. Figure 2b-2d demonstrated that PS-COOH MNPs were captured within the porous aerogel at multiple hierarchical levels, including adsorption onto the external surface (macro-surface), entrapment within the interconnected pores (meso/micropores), and binding along the internal nanofibers (nanoscale fiber surfaces). Those SEM images indicate high adsorption efficiency of the Bi-CS-CNF-PDA aerogels. NTA analysis demonstrates the successful removal of MNPs after 240 min. In Figure 2e, the lower density of the green spots indicates the decreased particle concentration after adsorption compared to the initial concentration (red spots). The time-dependent adsorption capacity of PS-COOH MNPs (0.5 mg/mL) was analyzed to understand the adsorption kinetics. As shown in Figure 2f, the bidirectional aerogel exhibits the highest adsorption capacity among the three aerogel samples with different microstructures, likely due to the enhanced binding affinity and larger specific surface area. As mentioned above, the fish rake-inspired bidirectional porous architecture further provides rapid and multiple water transport channels, resulting in higher water flux and faster diffusion of MNPs into the pores. This is consistent with the Kozeny-Carman equation (equation 4),^39^ which is a valuable formulation for describing the liquid transport through porous media:

4 $$k=\sqrt{\frac{\Phi^{2}D^{2}\phi^{2}\rho_{s}A_{s}\gamma \;\cos\theta }{75\eta \left(1-\phi \right)}}$$

where k is the spreading rate of liquid in porous materials; Φ is the sphericity of the particles in the porous materials; *D* is the diameter of the related spherical particle; ϕ indicates the aerogel’s porosity; ρ_*s*_ is the density of the solid material; *A*_*s*_ represents the specific surface area of the aerogel; γ is the surface tension of the liquid in the air; θ is the contact angle between the liquid and the solid surface; and η is the viscosity of the liquid. According to the equation, the spreading rate will improve when the specific surface area and porosity are increased. Moreover, the adsorption process is better described by the pseudo-first-order model (R^2^ = 0.9714), indicating that the adsorption rate of PS-COOH on the CS-CNF-PDA aerogel was dominated by physisorption and limited by diffusion or mass transfer steps. The adsorption capacity of the bidirectional foam increases dramatically during the first 60 min and then slows down, eventually reaching an equilibrium capacity of 393.53 mg/g after 240 min at 298 K, which is 9.37 and 2.3 times that of the foams with random and vertical porous orientation, respectively. Adsorption isotherms of the Bi-CS-CNF-PDA aerogel were also explored. As shown in Figure 2g, the PS-COOH MNPs adsorption capacity improves with the increase in the initial MNPs concentration from 0.05 to 2 mg/mL, and the foam maintains a stable adsorption capacity of ∼ 1200 mg/g at a concentration of 1.5 mg/mL. The data were fitted to the linear Langmuir and the Freundlich models (Figure 2h and 2i). The adsorption behavior is better explained by the Freundlich (R^2^ = 0.9774) than the Langmuir model (R^2^ = 0.4930) at 298 K. Meanwhile, the q_e_ obtained from the Freundlich nonlinear fit is very close to the experimental results, as shown in Figure 2j. The expression of the Freundlich model is indicated as equations 5 and ([Disp-formula eq6]).^40^

5 $$q_{e}=K_{F}{C_{e}}^{1/n}$$

6 $$\ln q_{e}=\frac{1}{n}\ln C_{e}+\ln K_{F}$$

where C_e_ (mg/L), q_e_ (mg/g), 1/n, and K_F_ represent the equilibrium concentration, the equilibrium adsorption capacity, the anisotropy factor, and the adsorption constant, respectively. The Freundlich isotherm describes adsorption on energetically heterogeneous surfaces, where active sites possess varying affinities toward the adsorbate. The Freundlich linear model provides a better fit to the PS-COOH adsorption process onto bidirectional aerogels, suggesting multilayer adsorption occurs predominantly on nonuniform surfaces enriched with active sites, rather than strictly one-to-one adsorbate–adsorbent interactions. This finding is consistent with the SEM results in Figure 2b-2d.

**Figure 2:**
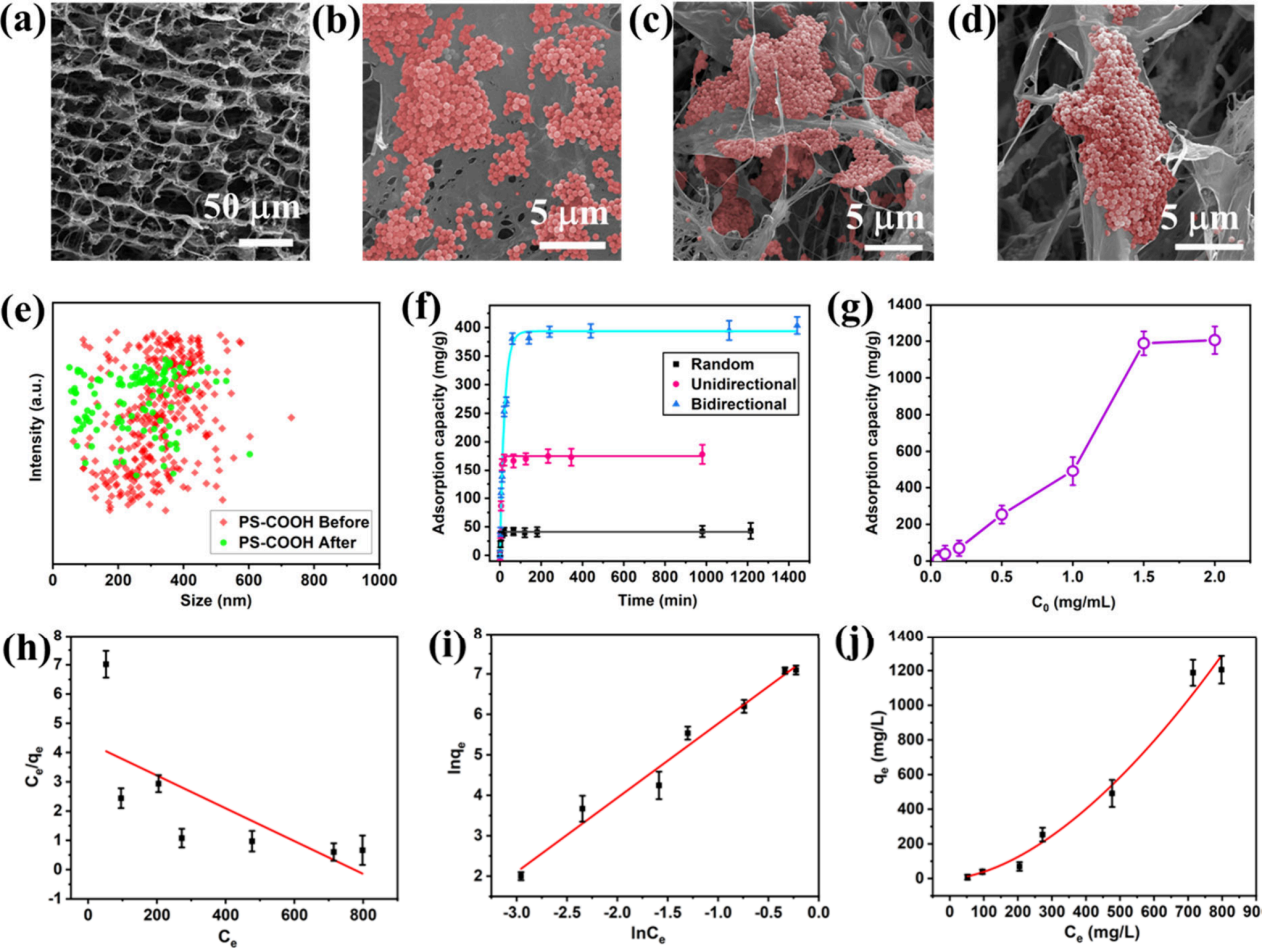
Adsorption performance of the synthesized Bi-CS-CNF-PDA aerogel for PS-COOH MNPs. (a–d) SEM images of the Bi-CS-CNF-PDA after adsorbing PS-COOH MNPs. (e) NTA analysis of the PS-COOH suspension before and after adsorption. (f) Adsorption capacities of random, unidirectional, and bidirectional CS-CNF-PDA aerogels for PS-COOH adsorption at different times. (g) Adsorption isotherm of PS-COOHs on the Bi-CS-CNF-PDA. Adsorption isotherms fitted by (h) the Langmuir and (i) the Freundlich models for the adsorption of PS-COOHs on the Bi-CS-CNF-PDA. (j) Adsorption isotherm of PS-COOH fitted by the Freundlich model. Error bars represent the standard deviation of three independent measurements.

### 3.3 Adsorption Performance for Various MNPs

The pH value has a critical effect on the PS-COOH adsorption performance of the Bi-CS-CNF-PDA aerogel, as it affects the surface charge properties of the foam. Figure 3a demonstrates the adsorption capacity of PS-COOHs by the bidirectional aerogel at different pH values. The maximum adsorption capacity was achieved at 393.53 mg/mL at a pH value of 7.0. When the pH values were adjusted to 5.0 and 3.0, the adsorption capacity decreased to 290.35 and 267.16 mg/g, respectively. It is mainly due to the attraction of H^+^ ions on PS-COOH MNPs in the solution, thereby decreasing the electrostatic attraction between the MNPs and the foam. When pH values increased to 9.0 and 11.0, the competition between OH^–^ and MNPs for adsorption on the foams resulted in lower PS-COOH adsorption capacities of 330.24 and 215.15 mg/g, respectively.

**Figure 3:**
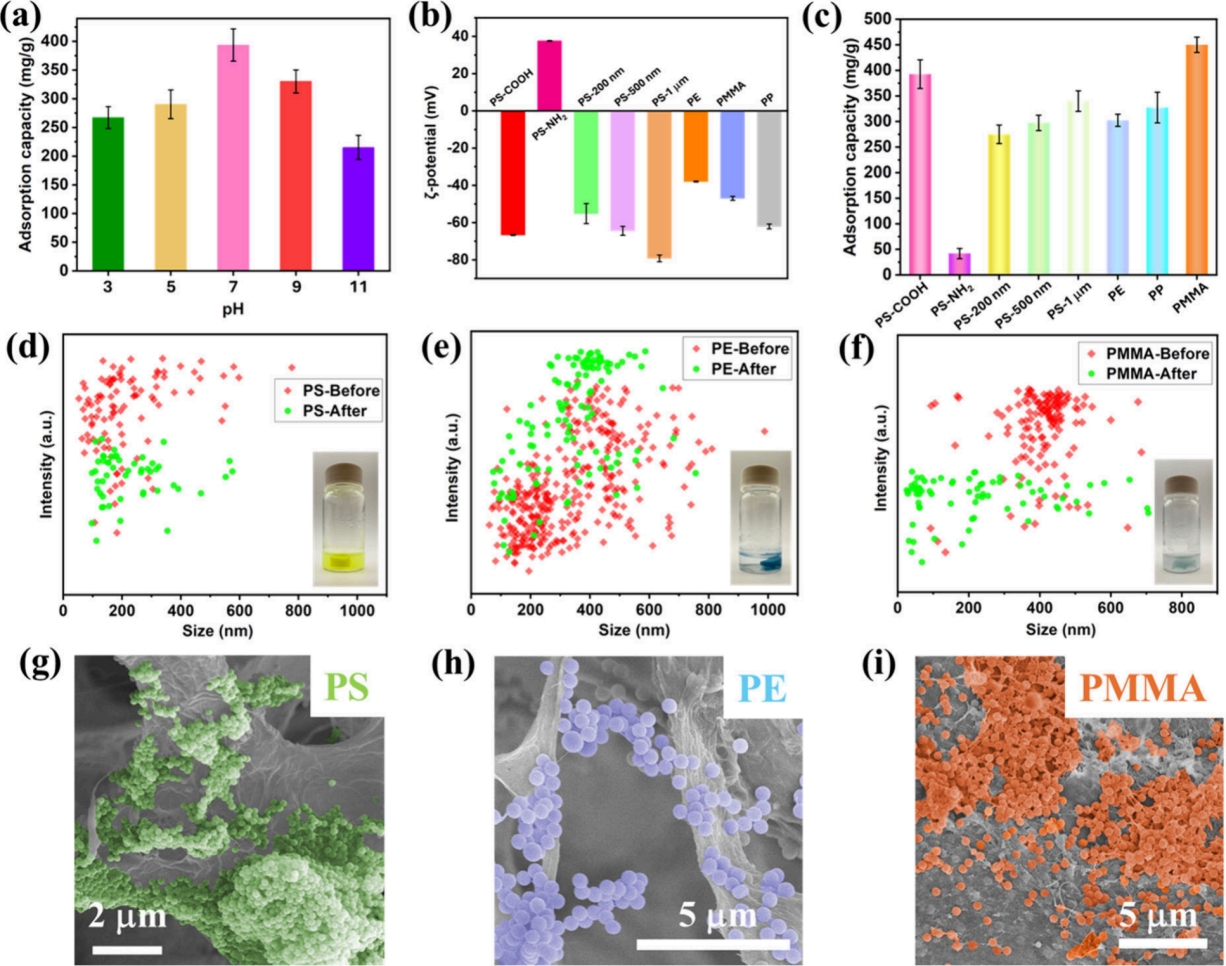
(a) Adsorption capacity of the synthesized Bi-CS-CNF-PDA for PS-COOH adsorption at different pH values. (b) Zeta potential of different MNPs. (c) Adsorption capacity of the synthesized Bi-CS-CNF-PDA for different MNPs. NTA of (d) polystyrene (PS), (e) polyethylene (PE), and (f) poly­(methyl methacrylate) (PMMA) MNP suspension before and after adsorption. SEM images of the synthesized Bi-CS-CNF-PDA after adsorbing (g) PS, (h) PE, and (i) PMMA MNPs. Error bars represent the standard deviation of three independent measurements.

To understand the effects of MNPs’ size and surface chemistry on adsorption, PS particles with different diameters (200, 500, 1000 nm) and PS-NH_2_ were used for adsorption tests through the Bi-CS-CNF-PDA. PS-NH_2_ and PS-COOH exhibited ζ potentials of 37.6 ± 0.21 mV and −66.7 ± 0.27 mV, respectively (Figure 3b), with corresponding adsorption capacities of 41.88 and 393.53 mg/g (Figure 3c). Since the surface of CS-CNF-PDA is positively charged in the water, its adsorption capacity for oppositely charged PS-COOH is much higher than that of PS-NH_2_, which illustrates the importance of electrostatic interactions between MNPs and the aerogel. Additionally, as shown in Figure 3c, the adsorption capacities for PS nanobeads with different diameters are 274.92 mg/g (200 nm), 297.19 mg/g (500 nm), and 340.05 mg/g (1000 nm). The results illustrate that the PS adsorption capacity of the foam increases with the diameter of the PS nanoparticles, mainly due to the enhanced negative surface ζ potential of larger particles. Additionally, the Bi-CS-CNF-PDA aerogel exhibits excellent adsorption performance for polyethylene (PE) and poly­(methyl methacrylate) (PMMA) nanoparticles. The adsorption capacity of the aerogel for PE, PP, and PMMA is as high as 302.38, 327.25, and 450.23 mg/g, respectively. The excellent absorbability of the Bi-CS-CNF-PDA toward various MNP species results from multiple types of adsorption interactions, which are enabled by abundant hydrogen-bonding sites on chitosan and cellulose, π-π interaction with PDA, electrostatic interaction involving the -NH_3_^+^ group, and vdW interactions. NTA analysis shown in Figure 3d-f exhibits a significant reduction in the concentration of PS, PE, and PMMA MNPs after their adsorption by the aerogels, respectively. Furthermore, SEM images in Figure 3g-3i validate the adsorption of PS, PE, and PMMA nanoparticles within the pore wall of the synthesized Bi-CS-CNF-PDA aerogels. A comparison of the previously reported biomass-based adsorbents for MNPs removal is shown in Table S1, where various parameters are compared such as MNP size, adsorption capacity, removal efficiency, and recyclability.^22,23,41–45^ In summary, the results demonstrate that the sustainable Bi-CS-CNF-PDA aerogels possess great potential for efficient remediation of MNPs in aquatic environments.

### 3.4 Adsorption Mechanisms Analysis by MD Simulation

Snapshots of each adsorption system within a unit cell in BIOVIA after optimization are exhibited in Figure 4a-f, indicating the most energetically favorable configurations for aerogel and MNPs adsorption. From the configurations, all six different MNPs are well adsorbed onto the Bi-CS-CNF-PDA aerogel molecules, where the PS, PS-COOH, and PMMA molecules are interpenetrated with polysaccharide chains, resulting in relatively larger contact areas. In contrast, the PS-NH_2_, PE, and PP molecules exhibit weaker degrees of binding with smaller contact areas. Through binding energy calculations, the binding energies of all MNPs are displayed in Figure 4g. The total binding energies of PS, PS-COOH, and PMMA are −38.94, −69.38, and −75.38 kcal/mol, respectively, while PS-NH_2_, PE, and PP possess the lower binding energies of −25.87, −11.29, and −21.25 kcal/mol, respectively. The results are consistent with the configurations in Figure 4a-f and the experimental results of adsorption capacities in Figure 3c. The CS-CNF-PDA networks adsorb PS-COOH and PMMA more firmly due to the formation of extra hydrogen bonds between MNPs and the polymer chains of aerogels. Figure 4h shows the energy components for each system, indicating that the binding energy of PS-COOH is dominated by electrostatic interactions (−47.51 kcal/mol) due to its improved polarity and electron density. In contrast, those of PS, PS-NH_2_, PE, PP, and PMMA are predominantly governed by vdW interactions (−31.80, −20.14, −10.80, −19.55, and −50.27 kcal/mol, respectively). In comparison, the nonpolar molecules and low surface electron density of PE, PP, and PMMA render their limited electrostatic interactions and hydrogen bonding. Thus, vdW interactions mainly contributed to the adsorption of those MNPs. The equilibrated configurations obtained from MD simulations are visualized using Discovery Studio, where the Interaction Monitor module was employed to identify and classify the types of interactions formed between the functional groups in the system. In addition to the regular hydrogen bonds between oxygen and hydrogen (Figure 4i­(i) and 4i­(ii)), weak hydrogen bonds π---HO and π---sigma (alkene, Figure 4i­(v))^46,47^ were also found to contribute to the interactions between PS-COOH and the aerogel, where abundant phenyl groups also indicate strong affinity with CS and CNF. Specifically, the presence of PDA offers more accessible binding sites for PS and PS-COOH MNPs to interact with the aerogel, such as π-π interaction and π-alkyl interaction (arising from vdW dispersion between aromatic π electrons and alkyl carbons) in Figure 4i­(iv) and 4i­(vi), respectively, further enhancing their binding strength with each other. Overall, the MD simulation reveals that electrostatic and vdW interactions, particularly hydrogen bonding and π-π interactions, play dominant role in the MNPs adsorption process.

**Figure 4:**
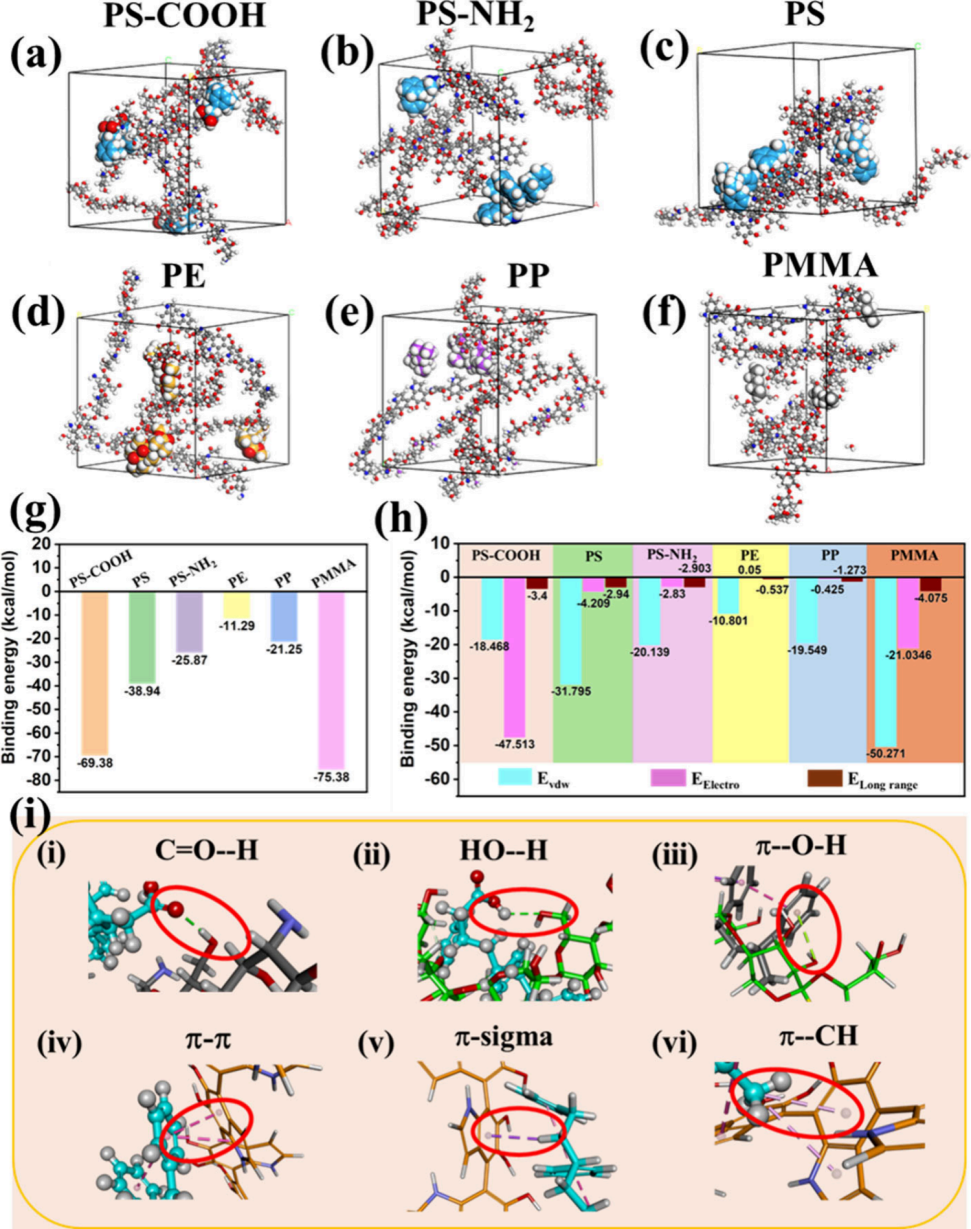
(a–f) Snapshots of steady configurations of the MNPs-aerogel adsorption system from MD simulations. (g) Binding energies between the CS-CNF-PDA aerogel and different MNPs. (h) Energy components of each adsorption system. (i) Visualized different types of interactions between PS-COOH and CS-CNF-PDA.

### 3.5 Adsorption Performance with a Benchtop Continuous-Flow Water Treatment System

To fully leverage the potential purification capability of the synthesized Bi-CS-CNF-PDA aerogels for MNPs, a benchtop continuous-flow adsorptive filtration-based system was developed, as illustrated in Figure 5a. In this system, the aerogel was fixed in a glass column, and synthetic MNP-contaminated water was pumped to flow through the column. After filtration, the effluent was pumped back into the adsorptive filtration column continuously for subsequent cycles. During the processing, adsorption and filtration effects act synergistically and simultaneously, greatly enhancing the purification efficiency of the aerogel. Briefly, 1 L PMMA containing water (20 mg/L, 500 nm) was treated by our closed-loop system as a case study. After four cycles, the blue water became clear after adsorptive filtration, as shown in Figure 5b. The influent and effluent solutions were analyzed using a fluorescence microplate reader to assess the removal efficiency of the MNPs. In Figure 5c, after the fourth cycle, the fluorescent intensity of the effluent at 560 ∼ 630 nm was similar to that of pure water, indicating the effective purification of PMMA-contaminated water after treatment and suggesting a 98% removal efficiency. Additionally, PS-COOH and PS MNPs were also tested by the system, and the removal efficiency of all particles was over 96% after four purification cycles, as shown in Figure 5d. Furthermore, a mixture of MNPs-containing water with PS-COOH, PS, PP, PE, and PMMA MNPs was combined in 1 L of water with a concentration of 20 mg/L. To track the MNPs in the aqueous suspension, a fluorescence microscope was used to examine both the influent and effluent. The fluorescence intensity of the effluent in Figure 5f decreased dramatically compared to that of the influent in Figure 5e, illustrating the efficient removal effect of MNPs through our water treatment column. The SEM image of the aerogel after adsorptive filtration further demonstrated that the packing aerogel was fully adsorbed by MNPs (Figure 5g), validating highly efficient water purification. Moreover, this system has also been applied to real lake water to further investigate its broad operational compatibility (Figure S4a). Postfiltration microscopic analysis indicates that the majority of suspended impurities and microorganisms present in the lake water were eliminated, as shown in Figures S4b and S4c.

**Figure 5:**
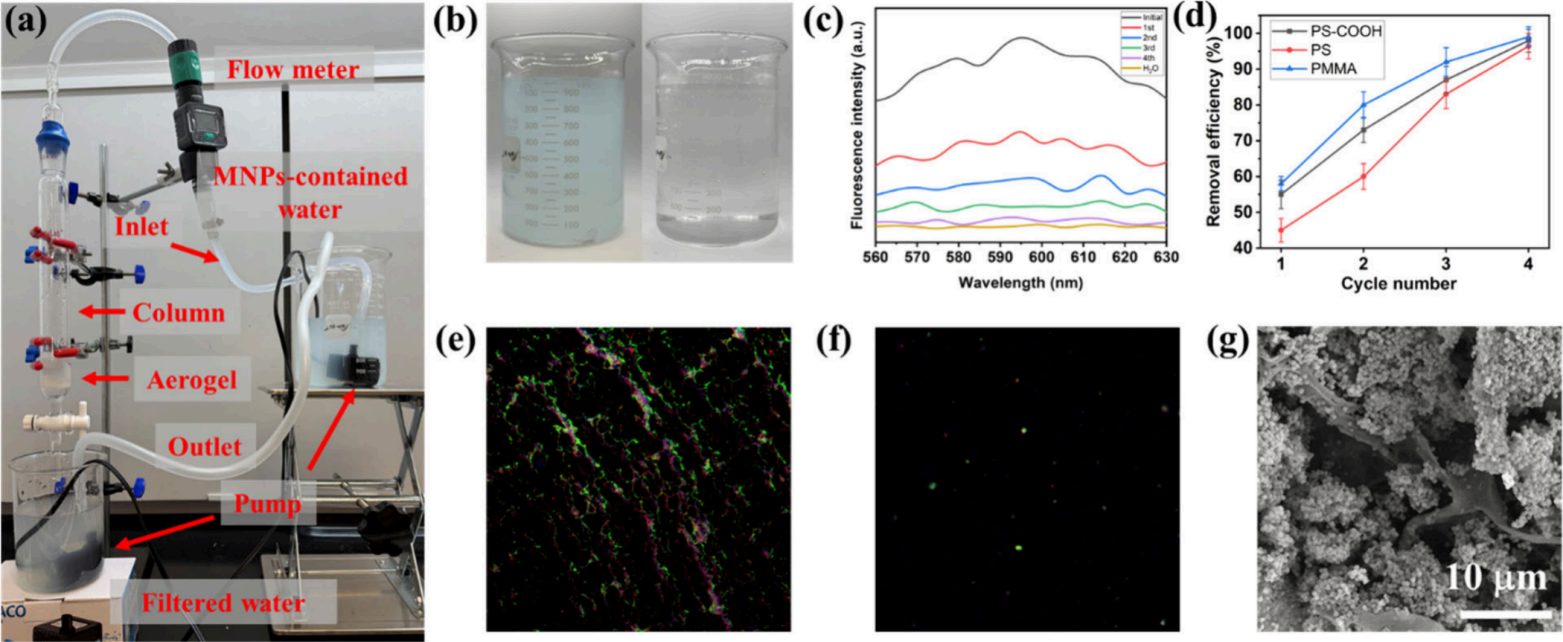
(a) Experimental setup for a benchtop continuous-flow adsorptive filtration water treatment system. (b) Images of a PMMA MNP suspension before and after treatment. (c) Fluorescence intensities of the influent and effluent suspensions after different treatment cycles of PMMA MNPs. (d) Removal efficiency of various MNPs after different treatment cycles. Fluorescence images of the MNP mixture suspension (e) before and (f) after adsorptive filtration. (g) SEM image of the Bi-CS-CNF-PDA aerogel covered with diverse MNPs. Error bars represent the standard deviation of three independent measurements.

Overall, the fabrication process of the Bi-CS-CNF-PDA aerogels involves naturally abundant and low-cost raw materials (chitosan, cellulose nanofibers, and low dosage polydopamine) and simple freeze-casting followed by freeze-drying, which can be easily scaled up. The estimated material cost is approximately $0.039/cm^3^ as shown in Table S2, indicating high economic feasibility for large-scale production. Moreover, the entire synthesis uses water as the only solvent without toxic reagents, making it environmentally benign, and the column-based adsorptive filtration can be completed by gravity-driven flow without other external pressure. Considering their low density and high adsorption capacity, the aerogels offer a low-cost, sustainable, and scalable solution for real-world wastewater purification.

### 3.6 Antibacterial Property Characterization

During MNP adsorption, the antifouling properties of the adsorbents, especially their antibacterial activity, are critical for preserving the aerogel’s structural integrity and operational lifespan. Nevertheless, few studies to date have reported the antibacterial properties of aerogels/sponges as adsorbents for MNPs in aquatic environments.^48,49^ It was proposed that the antibacterial activities of CS increase as the pH decreases from 7.0 to 5.0, where more -NH_3_^+^ residues form and bind with bacterial cells, thereby causing structural instability.^50^ Therefore, the antibacterial properties of Bi-CS-CNF-PDA were investigated in *E. coli* suspension at pH 6.0. Meanwhile, the two control groups without aerogel were set at pH 7.3 and 6.0, as shown in Figure S5a. The growth curves of *E. coli* are shown in Figure S5b. The OD_600_ of bacterial cultures decreases with decreasing pH value in the control groups. It is because the growth environment for *E. coli* deviates from the optical pH (7.0–7.4) and acidic stress suppresses the metabolic activity of *E. coli.* More importantly, the OD_600_ of bacterial culture significantly decreases from the aerogel group at pH 6.0, as reflected by the OD_600_ value at each time interval. It indicates the amount of *E. coli* decreases with the presence of CS-CNF-PDA aerogel. The antibacterial result is consistent with the previous study of CS-CNF aerogel microfibers^51^ in *E. coli* suspension. The excellent antibacterial properties are probably attributed to the polycationic nature of CS. CS interacts with the bacterial cell surfaces, disrupting membrane permeability and inducing leakage of intracellular constituents, such as glucose and lactate dehydrogenase (LDH), ultimately leading to cell death.^52^ The optical microscopy results are shown in Figures S5c and S 5d. After 6 h of culturing, the diluted bacterial suspensions of the Control sample (pH 6.0) and Aerogel sample (pH 6.0) were observed using an optical microscope. The control suspension in Figure S6c (white points) indicates a uniform dispersion of bacteria within the culture medium, with live cells (green) predominating over dead ones (Figure S5e). However, the number of bacteria cultured in the aerogel suspension was significantly lower than that in the pure medium, as shown in Figure S 5d. Additionally, the number of dead bacteria (red) increased substantially compared to the control sample, as illustrated in Figure S 5f. The results further demonstrate that the CS-CNF-PDA aerogel has an antibacterial effect in acidic conditions.

The performance longevity of the aerogel was assessed under realistic environmental conditions. After being immersed in natural lake water for 1 week, the aerogel maintained its structural integrity without visible biological fouling on the surface (Figure S6a). SEM images of the aerogels further exhibited strong MNP adsorption capacity in raw wastewater obtained from a local treatment facility (Figure S6b and 6c), highlighting its robustness and potential for practical water treatment applications.

## 4 Conclusions

In this work, fish-gill-inspired bidirectionally porous aerogels were developed for the first time using natural polysaccharides, including CS, CNF, and PDA. The hydrophobic and bidirectionally structured CS-CNF-PDA aerogels exhibited excellent MNPs adsorption performance, reaching over 300 mg/g from aquatic environments, including PS-COOH, PS, PE, PP, and PMMA. Mainly attributed to the bidirectional orientation, the porous aerogels exhibited an enhanced adsorption capacity for PS-COOH of over 390 mg/g, which was more than 9 times that of randomly oriented aerogels. More studies from MD simulations indicated that the excellent removal performance of MNPs is due to multiple intermolecular interactions, including vdW interactions, electrostatic interactions, and other intermolecular forces, such as hydrogen bonding, π–π stacking, and hydrophobic interactions. Additionally, the benchtop continuous-flow water treatment system demonstrated the potential of our materials to purify wastewater on a large scale with reliable removal efficiency. Finally, benefiting from its surficial characteristics, the sustainable structured aerogel exhibited excellent antibacterial properties against *E. coli* cells under acidic conditions. The mechanistic insights into the adsorption of MNPs laid the groundwork for a rational design of next-generation 3D solid-phase adsorbents and their integrated remediation systems for emerging contaminants in complex aquatic environments.

## Supplementary Material



## Notes

Compressive tests of aerogels with different orientations; energy profile of the PDA@CS-CNF composite in MD simulation; size distribution and standard curve of PS-COOH fluorescent nanoparticles; optical images of lake water after purification by Bi-CS-CNF-PDA; antibacterial tests of Bi-CS-CNF-PDA aerogels in *E. coli* suspension; SEM images of Bi-CS-CNF-PDA aerogels after MNP adsorption in raw wastewater; comparison of different biomass-based adsorbents for MNP removal; estimated materials cost for CS-CNF-PDA fabrication (PDF)
