# Supplementary material for: Fish Gill-Inspired Bidirectional Porous Polysaccharide Aerogels for Micro/Nanoplastics Removal
Source: ACS Appl Mater Interfaces. 2025 Nov 11;17(46):63488–99. doi: 10.1021/acsami.5c18203 (PMC12635965; doi:10.1021/acsami.5c18203)
Supplement: Supplementary file 1 [file am5c18203_si_001.pdf]

Supporting Information

Fish Gill-inspired Bidirectional Porous Polysaccharide Aerogels for Micro/Nanoplastics Removal

Yunchong Yang[1], Weijia Yan[2], Jun Ma[3], David Carmona[4], Chunka Zhou[2], Elise Nguyen[2], Jingjing Qiu[1,2]*

[1] Department of Materials Science and Engineering, Texas A&M University; 3003 TAMU, College Station, TX, 77843, USA

[2] Department of Mechanical Engineering, Texas A&M University; 3123 TAMU, College Station, TX, 77843, USA

[3] Department of Biomedical Engineering, Texas A&M University; 3131 TAMU, College Station, TX, 77843, USA

[4] Department of Mechanical Engineering, Texas A&M University at Qatar; PO Box 23874, Education City, Doha, Qatar

*Corresponding author

*Email address: Jennyqiu@tamu.edu

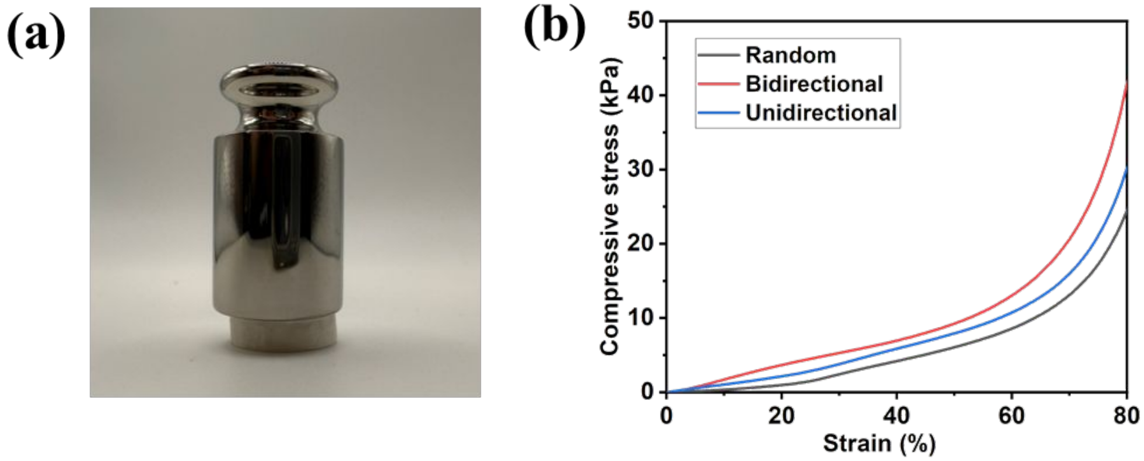

Figure S1. (a) Digital photo of Bi-CS-CNF-PDA with a 100 g load. (b) Compressive stress-strain curves of three aerogels with different orientations.

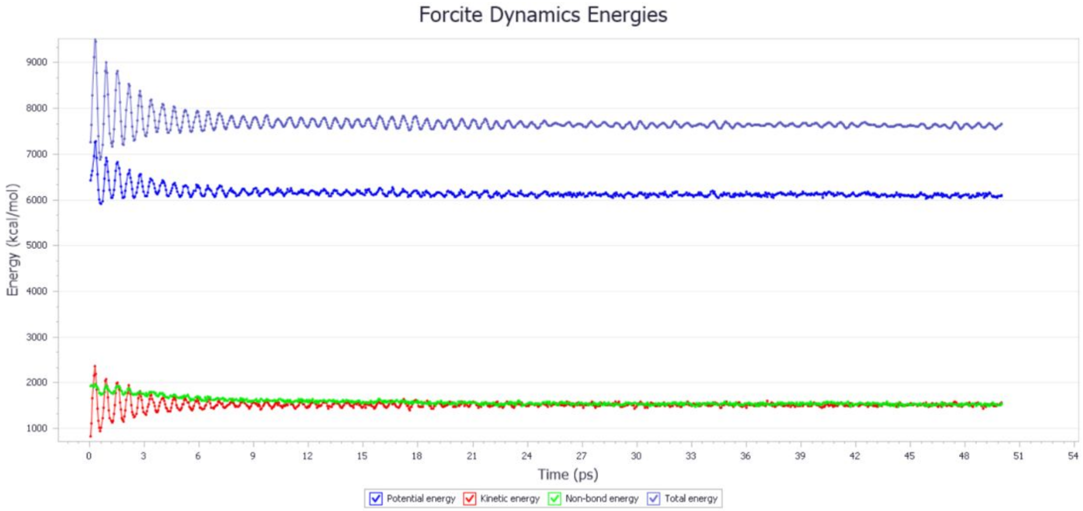

Figure S2. Energy profile of the CS–CNF–PDA composite during MD simulation.

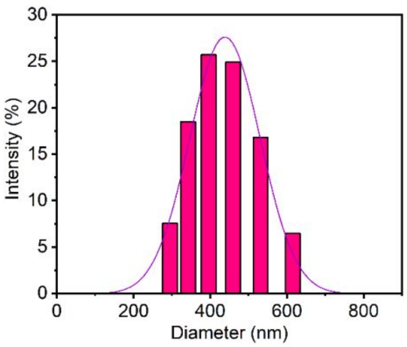

Figure S3. Size distribution of PS-COOH MNPs.

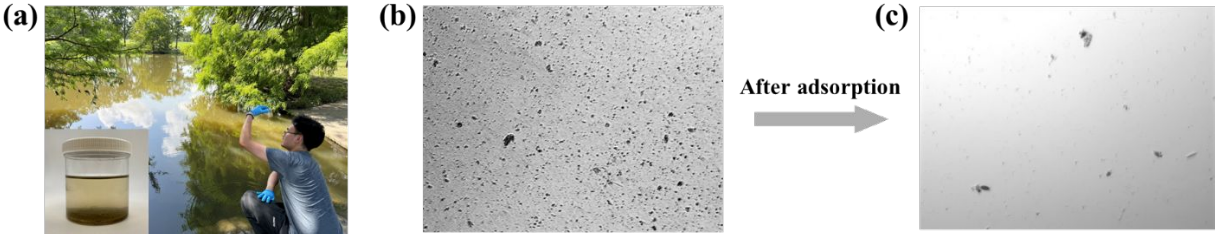

Figure S4. (a) Photo image of the water sample from lake. (b) and (c) Optical microscope images of the lake water before and after purification, respectively.

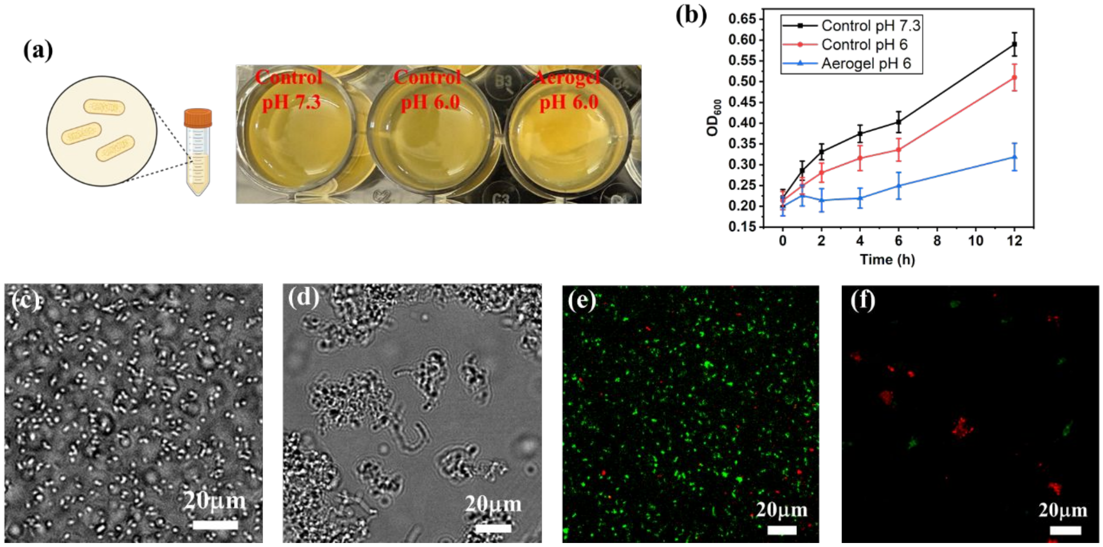

Figure S5. (a) Experimental groups for antibacterial test. (b) Growth curves of *E. coli* in different conditions. Optical microscopy of diluted *E. coli* suspension from the sample (c) Control pH 6 and (d) Aerogel pH 6. Fluorescent live and dead *E. coli* cells from the suspension in (e) Control pH 6 (f) Aerogel pH 6.

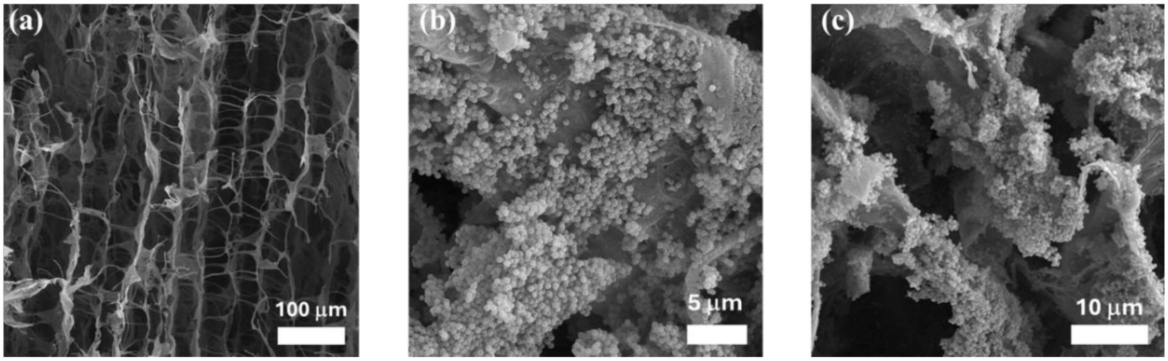

Figure S6. (a) SEM image of the Bi-CS-CNF-PDA aerogel after immersed in lake water for one week. (b) and (c) SEM images of the Bi-CS-CNF-PDA aerogel after adsorption of MNPs in raw wastewater.

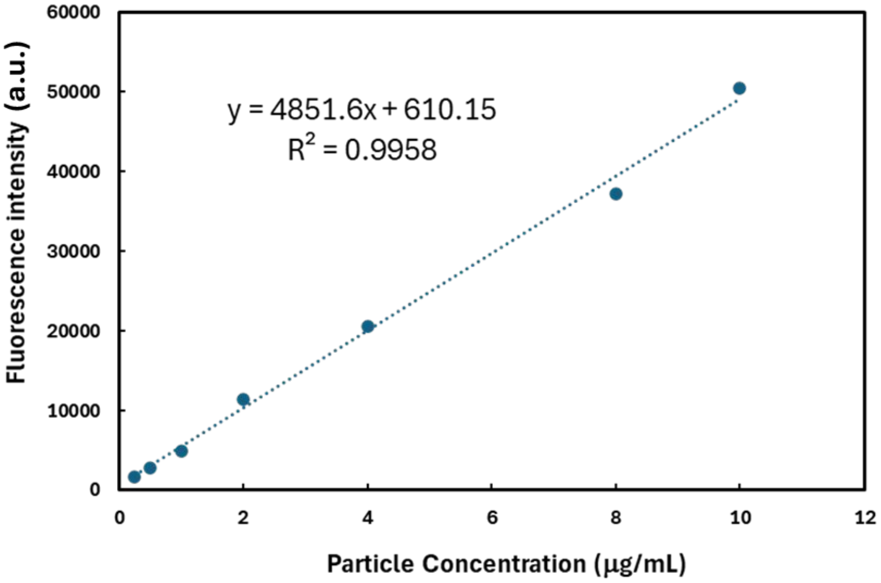

Figure S7. The linear relationship between the fluorescent intensity of PS-COOH nanoparticles and concentration.

Table S1. The comparison of previously reported biomass-based adsorbents for micro/nano-plastics removal.

Materials	Size of plastics	Adsorption capacity (mg/g)	Removal efficiency (%)	Recyclability (cycles)	Ref.
Starch-gelation sponge	5 μm	20.43 (PMMA)<br>21.79 (PS)	90% (PMMA)<br>80% (PS)	2	[1]
Lysozyme amyloid fibrils	500 nm	-	93.4% (PS-COOH)	-	[2]
Oat protein sponges	1 μm	10.87	81.2% (PS)	4	[3]
Chitin/GO/<br>Chitosan	1 μm	9.74 (PS)<br>7.83 (PS-COOH)<br>12.4 (PS-NH_2)	92.1% (PS)<br>81.3% (PS-COOH)<br>87.1% (PS-NH_2)	3	[4]
PEI/Cellulose fibers	110-150 nm	0.80 (PMMA)<br>0.86 (PVC)<br>0.84 (PVAc)	95% (PMMA)<br>98% (PVC)<br>98.8% (PVAc)	-	[5]
Fe_3O_4/PDA/CS	28-37 μm	125 (PET)<br>130 (PE)<br>127 (PS)	91.6% (PET)<br>94.6% (PE)<br>92.3% (PS)	3	[6]
Acinetobacter sp. biofilm	400-500 nm	-	87.2% (PS)<br>60.5% (PS-COOH)<br>14.0% (PS-NH_2)	-	[7]
PDA@CS-CNF	200 nm-1 μm	340.05 (PS); 393.53 (PS-COOH); 302.38 (PE); 327.25 (PP); 450.23 (PMMA)	96.4% (PS); 98.5% (PS-COOH); 99.4% (PMMA)	4	**This work**

Table S2. Estimated material cost for the Bi-CS-CNF-PDA aerogel.

Raw materials	Unit price ($/g)	Weight used (g)	Cost ($)	Average cost ($/cm^3)
Chitosan	2.41	0.01	0.0241	$\frac{0.04994\ \$}{1.25\ \text{cm}^3} = 0.039$
Cellulose nanofiber	1.60	0.01	0.016	
Dopamine hydrochloride	4.92	0.002	0.00984	
Total	-	-	0.04994	

References

[1] Fu, J.; Liu, N.; Peng, Y.; Wang, G.; Wang, X.; Wang, Q.; Lv, M.; Chen, L. An ultra-light sustainable sponge for elimination of microplastics and nanoplastics. *J. Hazard. Mater.* **2023**, *456*, 131685.

[2] Peydayesh, M., Suta, T., Usuelli, M., Handschin, S., Canelli, G., Bagnani, M. and Mezzenga, R. Sustainable removal of microplastics and natural organic matter from water by coagulation–flocculation with protein amyloid fibrils. *Environ. Sci. Technol.* **2021**, *55* (13), 8848-8858.

[3] Wang, Z., Sun, C., Li, F. Chen, L. Fatigue resistance, re-usable and biodegradable sponge materials from plant protein with rapid water adsorption capacity for microplastics removal. *Chem. Eng. J.* **2021**, *415*, 129006.

[4] Sun, C., Wang, Z., Zheng, H., Chen, L. and Li, F. Biodegradable and re-usable sponge materials made from chitin for efficient removal of microplastics. *J. Hazard. Mater.* **2021**, *420*, 126599.

[5] Batool, A; Valiyaveettil, S. Surface functionalized cellulose fibers–A renewable adsorbent for removal of plastic nanoparticles from water. *J. Hazard. Mater.* **2021**, *413*, 125301.

[6] Zheng, B., Li, B., Wan, H., Lin, X., Cai, Y. Coral-inspired environmental durability aerogels for micron-size plastic particles removal in the aquatic environment. *J. Hazard. Mater.* **2022**, *431*, 128611.

[7] Kim, B., Lee, S.W., Jung, E.M. and Lee, E.H. Biosorption of sub-micron-sized polystyrene microplastics using bacterial biofilms. *J. Hazard. Mater.* **2023**, *458*, 131858.
